# Prospects and limitations of e-learning application in private tertiary institutions amidst COVID-19 lockdown in Nigeria

**DOI:** 10.1016/j.heliyon.2020.e05457

**Published:** 2020-11-06

**Authors:** Wasiu Oyeleke Oyediran, Ayodeji Motunrayo Omoare, Maryam Adebusola Owoyemi, Abayomi Olatoke Adejobi, Rafiat Bolanle Fasasi

**Affiliations:** a Federal University of Agriculture, Abeokuta, Nigeria; b Federal College of Education, Abeokuta, Nigeria; c Tai Solarin College of Education, Ijebu-Ode, Nigeria

**Keywords:** Education, COVID-19, E-learning, Limitation, Lockdown, Pandemic, Prospect, Tertiary institutions, Nigeria

## Abstract

E-learning has numerous potentials to spur education development in tertiary institutions in Nigeria. It impacts positively on the educational process, unlike the physical chalkboard in the classrooms. The outbreak and fast spread of the COVID-19 led to the closed down of schools. Efforts to revamp education due to prolong lockdown made the government enforce e-learning in tertiary institutions across the country. It is however worthy to know that these directives did not make much change as a result of poor infrastructure and networking. Hence, this study investigated compliance with e-learning during COVID-19 pandemic lockdown by the instructors in the private tertiary institutions in Nigeria vis-à-vis their socio-economic factors and limitations encountered. A systematic sampling technique was adopted to select 180 respondents from the staff list. A validated questionnaire was used to collect data on socio-economic variables (SEV), compliance (ϒ) to e-learning, and limitations (Ls) while multiple linear regression model (R) was used to test the interaction between the compliance and limitations. Results show that age (β = 0.351), educational attainment (β = 0.843) and teaching experience (β = 0.169) influence e-learning compliance at p < 0.05. It was also found that 67.3% compliance with e-learning took place in the Universities compared to 59.1% in the Polytechnics and 52.8% in the Colleges of Education. Regression shows that constraints affected the level of compliance (R^2^ = 0.73). The study concludes that constraints are major obstacles to the compliance and prospects of e-learning in the Private Tertiary Institutions in Nigeria.

## 1 Introduction

There is a pervasive crisis in Sub-Saharan Africa's teaching and learning development systems. The high level of illiteracy and poor infrastructure causes set back to educational development. The recent coronavirus outbreak (COVID-19) compounded to the problem and has taken tolls on all socio-economic sectors without exception to the educational system in Nigeria. During the lockdown, many female students have become victims of rape which have led to unwanted pregnancies, and cases of death also reported. For instance, a female undergraduate student of Laboratory Technology Department, Federal College of Animal and Production Technology, Moor Plantation Ibadan, Oyo State was raped to death (Ajayi, 2020); equally incident of gang-raped and death of a female undergraduate student, University of Benin, Benin City, Edo State was reported (Adejumo, 2020); and another rape and murder case of a postgraduate student of University of Ibadan occurred during the pandemic (Omonobi et al., 2020).

Besides, the lockdown exposed the nation's poor health infrastructure, caused economic depression, and worsened the unemployment and insecurity situation in the country. Banditry, kidnapping, robbery, and Boko-Haram terrorist attacks are on the rampage. From the National Centre for Disease Control report, the affected people increased from 407 to 48,569 with 1,098 deaths from February to September 20, 2020 (Nigeria Centre for Disease and Control, 2020). The pandemic has led to the total closedown of all schools from primary to tertiary levels which makes students becoming redundant at home. Report of Education in Emergency Working Group has also shown that about 46 million Nigerian students are affected by the schools' closure (EiEWG, 2020); this is very significant as it represents 25 percent of Nigerian total population. From the global perspective, the COVID-19 pandemic has made the largest devastative impact on the education sector and affected learners and teachers from pre-primary to the tertiary education level (Andreas, 2020). Universities closed their premises and countries shut down their borders in response to lockdown measures. Findings from 200 countries in the mid-April 2020 showed that 94 percent of learners were affected by the pandemic around the world, which represents 1.58 billion learners (United Nations, 2020). Additionally, UNESCO (2020) reported that the closure of higher institutions has affected over 91 percent of the students' population in the world and that 23.8 million students may drop out or not be able to secure admission to schools in the 2021 academic calendar.

Remote learning became a lifeline for education during the pandemic but, the opportunities that digital technologies offer go well beyond a stopgap solution during a crisis (Andreas, 2020). According to Eze et al. (2018), e-learning education is the all-inclusive blending of ICT gadgets and modern telecommunication equipment into the education system. Andreas (2020) and Eze et al. (2018) maintained that e-learning is a hallmark of distance learning. Digital technology offers entirely new answers to the question of what people learn, how they learn, and where and when they learn. Andreas (2020) further stated that technology enables teachers and students to access specialized materials well beyond textbooks, in multiple formats, and in ways that bridge time and space. Meanwhile, Eduard and Lucian (2020) hinted that e-learning is an innovative platform for transmitting knowledge and skills to the learners; it is cheap, saves time, has a wider coverage, and as well promoting team learning and collaboration. Andreas (2020) reiterated that technology promotes deep learning, and allows schools to respond better to the varying needs of the students.

In a bid to avoid brain-drain and prevent the total collapse of the education sector in the country, Nigeria joined other leagues of developed countries and incorporate e-learning in the education system. Although Nigeria Open University operates e-learning to deliver lectures and give assignments to the students this digitization has not been sufficiently harnessed in many tertiary institutions across the country. It is either the lecturers are not ICT-compliance or the students are disadvantaged. In some tertiary institutions where ICT is applied it is limited to students' registration and examination. Much effort has not been geared towards effective teaching and learning process and students’ academic performance through e-learning.

While COVID-19 has forced Nigeria to embrace e-learning to keep pace with rapid development in the area of technology the implementation is at a very low pace.

In advanced countries, the changes are eminent in the educational sector as traditional teaching methods have been transformed into modern methods (Kacerauskas and Kusaityte, 2020). Students in the College routinely learned and studied with technology in advanced countries.

For instance, the Chinese Ministry of Education introduced a *Suspending Classes Without Stopping Learning* policy to ensure that learning was not compromised at any time during the COVID-19 pandemic lockdown (Zhang et al., 2020), and provide flexible online learning to hundreds of millions of students from their homes (Huang et al., 2020). Online platforms were the most popular tool used during the COVID-19 pandemic in the OECD countries (Schleicher and Reimers, 2020). The instructional tools are designed in such a way that students could explore educational content at will while teachers delivered the lessons using virtual meeting platforms (Andreas, 2020). In Sweden, post-secondary schools have switched to mainly distance learning from the onset of the pandemic (UNESCO, 2020). In the online review conducted by Chaka (2020) in South Africa and the United State of America, it was found that during the COVID-19 lockdown 17 of the 21 South African universities and 63 of the 64 U.S. universities migrated to e-learning and utilized Zoom, Canvas, and Blackboard as the topmost online tools and resources. In March 2020, the Italian government equipped schools with digital platforms, trained school instructors on techniques for e-learning, and gave digital devices to poor students to cushion the effects of the COVID-19 pandemic (The Republic of Italy, 2020). In the same March 2020, Pakistan's Higher Education Commission (HEC) compelled higher institutions to commence e-learning. Also, teachers in Greece conducted virtual real-time classes in conjunction with other online learning tools (Ministry of Education and Religious Affairs, 2020; Schleicher and Reimers, 2020). Australia rapidly switched to online learning in the wake of the pandemic (Ali, 2020). This would prevent compromising education in a pandemic situation (The News, 2020).

In the Nigerian context, the number of students attending tertiary institutions outnumbered the schools’ infrastructure. The high cost of ICT accessories and inadequate resource persons are among the problems limiting e-learning in Nigeria (Adeoye et al., 2020). In Nigeria, many institutions find it difficult to conceptualize and implement e-learning initiatives locally.

Specific objectives are to:

i. examine the level of compliance of instructors to e-learning during the COVID-19 pandemic lockdown; and
ii. identify limitations to the use of e-learning in the selected private tertiary institutions.

### 1.1 Hypothesis

H_01_: Constraints have no significant influence on the instructors' compliance with e-learning in the selected tertiary institutions.

This hypothesis is premised on the assumption that constraints could affect the optimization of e-learning in Nigerian tertiary institutions. According to the United Nations (2020) report, some tertiary institutions jettisoned e-learning during school closure due to the lack of information technology (IT) infrastructure.

Firstly, the power supply in Nigeria is erratic, barbaric, and worrisome. It has become a national problem, very embarrassing as it affects all sectors in Nigeria (Oyediran and Dick, 2018; Adeoye et al., 2020). Part of the effort put in place by the government was the diversification of power source from hydroelectric to the use of gas to generate higher megawatt yet there is no significant improvement, it is even getting worst. The power generation in the country is abysmally low, about 1400 Megawatt (Thisday, 2016).

Secondly, ICT hardware to power e-learning is imported to Nigeria since the country has not been able to develop its local manufacturing industries. The cost of importation is very high going by the ever-rising foreign exchange rate which is as high as ₦380 to $1USD. This has led to inflation and escalating prices of ICT hardware. Computer accessories are becoming too expensive to buy and it becomes an impediment to e-learning. Adeoye et al. (2020) reported that the price of computer hardware and software is several times more expensive in Nigeria than in advanced countries.

Thirdly, the ICT experts are very scanty to the extent that the available ones have been over-stretched due to the high demand for their service. Also, the charges of expatriates are outrageous when consulted whereas the cost of international personnel training is highly exorbitant. Nigerian tertiary institutions cannot afford all these costs going by their meager fund allocated to them in the national budget. The budgetary allocation to education is as low as 4–7.24% of the annual budget in the last decade which contradicts the 15–20% recommended by UNESCO (Ameh and Aluko, 2019). This invariably affects manpower development and e-learning in Nigeria. Network administrators and local technicians to service and repair computer facilities do not receive any training at all (Adeoye et al., 2020).

Fourthly, the state of infrastructure is generally appalling in Nigeria. The lecture rooms are dilapidated, incomplete, and not conducive to effective learning. The laboratories, libraries, and ICT units are ill-equipped. Alternative power supply through a big generating set (MIKANO) is expensive to run in terms of fuelling, servicing, and repairing. Mahmood (2020) and Ali (2020) reported that poor state of infrastructure and manpower development affects the efficient use of the internet. High tariff significantly contributed to the high purchasing cost of ICT facilities which make it difficult for the government to institutionalize e-learning in many tertiary institutions.

### 1.2 Purpose of this study

Going by the rapid rising cases of COVID-19 in the country, the Federal Government of Nigeria locked down two states (Lagos and Ogun) where the index visited, and FCT Abuja while other affected states joined as the coronavirus spreads. Federal Ministry of Education enforces electronic learning in the tertiary institutions as a way to ensure the school system is not collapsed. Beyond the government pronouncement and swift shift to e-learning across the world, researchers have not empirically examined the influence of socio-economic variables of instructors and constraints on e-learning compliance during the COVID-19 pandemic. More so, the World Bank (2020b) is of the view that few pieces of research have been conducted on the scale of e-learning provision, compliance, and limitations in the higher institutions. Many studies focused on necessity of e-learning during lockdown (Ali, 2020), instructional strategies for online (Mahmood, 2020), level of preparedness for e-learning (Eduard and Lucian, 2020; EiEWG, 2020), e-learning and tertiary education experience (Adeoye et al., 2020), and use of online instruction, tools and resources during COVID-19 (Chaka, 2020). It is hereby imperative to investigate the SEV influence on e-learning compliance and pros and cons of e-learning strategy to strengthening Nigeria's educational system.

E-learning a technology-driven model and makes teaching take place without physical contact with the learners. The practical avenue to avoid drawback in the Nigerian education system during the COVID-19 is e-learning. E-learning supports knowledge and performance management (Mahmood, 2020; The World Bank, 2020a). According to Eduard and Lucian (2020), educational technology as a field of education or new terminology has been like teaching aids or apparatus. E-learning has offered tremendous opportunities for teaching by electronic means (Kacerauskas and Kusaityte, 2020; The World Bank, 2020a). Students that undertake electronic studies generally performed better than those in face-to-face courses. Andreas (2020) opined that the academic performance of learners that used the electronic approach supersedes those who studied the traditional approach. E-learning is a new learning model in Nigeria, with all its potentialities.

## 2 Research method

This study was carried out in the southwest, Nigeria. The southwest geo-political zone comprises of six states which are Ekiti, Lagos, Ogun, Ondo, Osun, and the Oyo States.

Kothari (2004) sample size determination formula was used to estimate the sample size to be selected for this study, the formula is:

$$\text{n }=\frac{{\text{z}}^{2}\text{pq}}{{\text{c}}^{2}}$$

At the confidence interval (c) of 5% and confidence level (z) of 1.96 for 95%, a 69% proportion of an attribute of the population (p), and 17% desired level of precision (q), the estimated sample size is 180.2. For ease of distribution, the sample size was approximated to 180.

A multi-stage sampling method was used for the selection of a representative sample. This sampling method is chosen because it is an advance of the principle of cluster sampling. The method is recommended for a big inquires extending to a considerable large geographical area (Kothari, 2004), like the case under study, private tertiary institutions in Nigeria. The merits of this method are that it is easier to administer than most single-stage designs, and a large number of units can be sampled for a given cost because of sequential clustering, whereas this is not possible in most of the simple designs. The three states randomly selected out of six states in the first stage are Lagos, Ogun, and Oyo. Private Universities, Polytechnics, and Colleges of Education in Lagos, Ogun, and Oyo the States, Nigeria were chosen for this study. There are five accredited private Universities in Lagos State, seven private Polytechnics, and five private Colleges of Education. Ogun State has eleven private Universities, four private Polytechnics, and two private Colleges of Education while Oyo State has six Private Universities, five private Polytechnics, and three private Colleges of Education. In the second stage, one University, one Polytechnic, and one College of Education were selected from each state; these gave rise to 3-Colleges of Education, 3-Polytechnics, and 3-Universities selected. In the third stage, a systematic sampling technique was adopted to select every 13^th^ name on the staff lists to arrive at twenty instructors per institution. Systematic sampling is spread more evenly over the entire population; it is an easier method of sampling and can be conveniently used even in the case of large populations (Kothari, 2004). Thus, 180 instructors were selected from the nine tertiary institutions. Government-owned institutions particularly Universities were on industrial action at the time of conducting this research so they are exempted.

The authors highly considered the issues of validity and reliability in the study. To ensure the validity of the study, the content validity of the instrument was carried out by experts in ICT and Education. Content validity according to (Dave, 2012; Wilson et al., 2012) is the extent to which a measure represents all facets of a given social construct. It is the most critical criterion and indicates the degree to which an instrument measures what it is supposed to measure (Kothari 2004). Similarly, the reliability of the instrument was carried out by the test re-test method. The coefficient of reliability was 0.79, an indication that the instrument is reliable.

This study adopted a survey method for the primary data collection on socio-economic variables, compliance, and constraints to e-learning in the private tertiary institutions. Respondents showed a willingness to provide answers to the questions contained in the questionnaire. This is a quite popular method of data collection. It does not give room for the interviewer's bias; answers are in respondents' own words hence the results can be made more dependable and reliable (Kothari, 2004). In the course of conducting this study, authors strictly adhered to all standards of ethical principles to safeguard the rights of respondents in terms of the respondents' autonomy, privacy, anonymity, and confidentiality. All procedures adopted in the conduct of this study followed ethical standards of the institution approved by the Institution Committee on Research (ICR) and Joint Technical Task Team on COVID-19 (JTTT), Ogun State, Nigeria on April 23rd, 2020 for the period of 3–5 months.

### 2.1 Analytical methods

Age and years of experience were measured at ratio level and converted to an interval level for presentation. Educational Attainment was measured as the number of years spent in the schools to obtain various qualifications by the respondents. Compliance (ϒ) with e-learning was conceptualized as Complete (3), Partial (2), and Not at all (1) for descriptive statistics and ANOVA.

Model Specification:

(1) n_1_(t.q_1_)+n_2_(t.q_2_)+n_3_(t.q_3_)…+n_i_(t.q_i_) = ϒ

where; t is the time taken to deliver the course online, q is the course taken, and n is the number of the times the course was taken.

(2) f_n_x_n_ = L_n_

f = frequency, x = score and L_n_'s referred to the problems confronting the adoption of e-learning such as poor electricity supply, high cost of e-learning facilities, and poor internet connectivity.

Multiple linear regression models determine the extent of variations to e-learning compliance among the instructors in the selected private institutions (See Table 1).

**Table 1:** Variable Choice and definition for the e-learning compliance.

	Variables	Description	Variable type	Expected relationship
**Dependent variable**				
ϒ	Compliance to e-learning	Scores	Continuous	Positive
**Independent variables**				
L_1_	Poor electricity supply	Scores	Continuous	Negative
L_2_	High cost and poor quality of e-learning facilities	Scores	Continuous	Negative
L_3_	The poor technical know-how of e-learning	Scores	Continuous	Negative
L_4_	Poor internet connectivity	Scores	Continuous	Negative
L_5_	Lack of telecommunication infrastructure	Scores	Continuous	Negative
L_6_	Lack of training support by the institutions	Scores	Continuous	Negative

α = Constant; and ei = error term.

According to Kothari (2004), the primary function of regression analysis is to determine the various factors which cause variations of the dependent variable. The functional form gives the best fit in terms of the high value of the R^2^, the low value of Durbin-Watson, the sign of coefficients, as well as better F-ratio (see Table 2).

(3) ϒ = f(L_s_)

ϒ = f (f_n_x_n_)

**Table 2:** Correlation between age of the respondents and e-learning compliance.

Model	R	R^2^	Adjusted R^2^	Std. Error of the Estimate	Durbin-Watson
1	0.351^a^	0.123	0.118	5103.326	1.458
**ANOVA**^b^					
	**Sum of squares**	**df**	**Mean Square**	**F-Statistics**	**Sig.**
Regression	6.520E8	1	6.520E8	25.034	0.024^c^
Residual	4.636E9	178	2.604E7		
Total	5.288E9	179			
**Coefficients**					
**Model**	**Unstandardized Coefficients**		**Standardized Coefficients**		**Sig.**
	β	Std. Error	Beta	T	
(constant)	4999.115	2159.177		2.315	0.022
Age	253.196	50.605	0.351	5.003	0.000^d^

Source: Field Survey (2020).

a Predictor: (Constant), age.

b Dependent variable: e-learning compliance.

c Predictor: (Constant), age.

d Predictor: (Constant), age.

Thus the explicit model is:

(4) ϒ = α + β_1_L_1_ + β_2_L_2_ + β_3_L_3_ + β_4_L_4_ + β_5_L_5_ ……+ β_n_L_n_ + ei

(5) ϒ = α + β_1_(f_1_x_1_) + β_2_(f_2_x_2_) + β_3_(f_3_x_3_) + β_4_(f_4_x_4_) + β_5_(f_5_x_5_) ……+ β_n_(f_n_x_n_) + ei

where ϒ is compliance with e-learning and β_n_'s referred to the parameter to be estimated.

## 3 Findings

### 3.1 Influence of selected socio-economic variables on e-learning compliance

**Figure 1:**
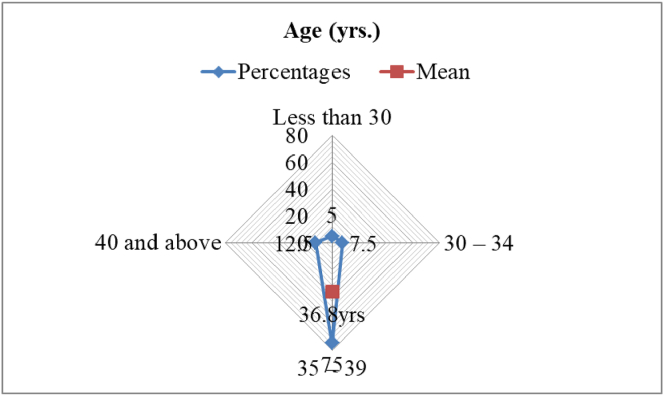
Radar showing the age distribution of the respondents. Source: Field Survey (2020).

Figure 1 portrays age categories of the respondents with seventy-five percent fell within 35–39 years while 12.5% were older than 40 years. The estimated average age was 36.8 years for the respondents.

Table 2 shows the regression results of the age of the respondents as a predictor of compliance with e-learning. The result indicates that there is a positive but weak correlation between the age of the respondents and compliance with e-learning (R = 0.351^a^ < 0.51 for 180 degrees of freedom). The significant of F-statistics (F = 25.034, p = 0.024^c^) indicates a linear relationship between the age and compliance to e-learning. The regression model explains that 12.3% variation in e-learning compliance was due to age (R^2^ = 0.123^b^) while 87.7% is due to the residual factors excluded from the model. A significant relationship was found between the age of the respondents (β = 0.351^d^) and e-learning compliance at p < 0.05 which is 35.1%. Therefore, age is a determinant of compliance with e-learning in the Nigerian Private Tertiary Institutions. The implication is that younger instructors should be the target of e-learning training and skills acquisition because they are easy to train and have a high tendency to comply with e-learning.

**Figure 2:**
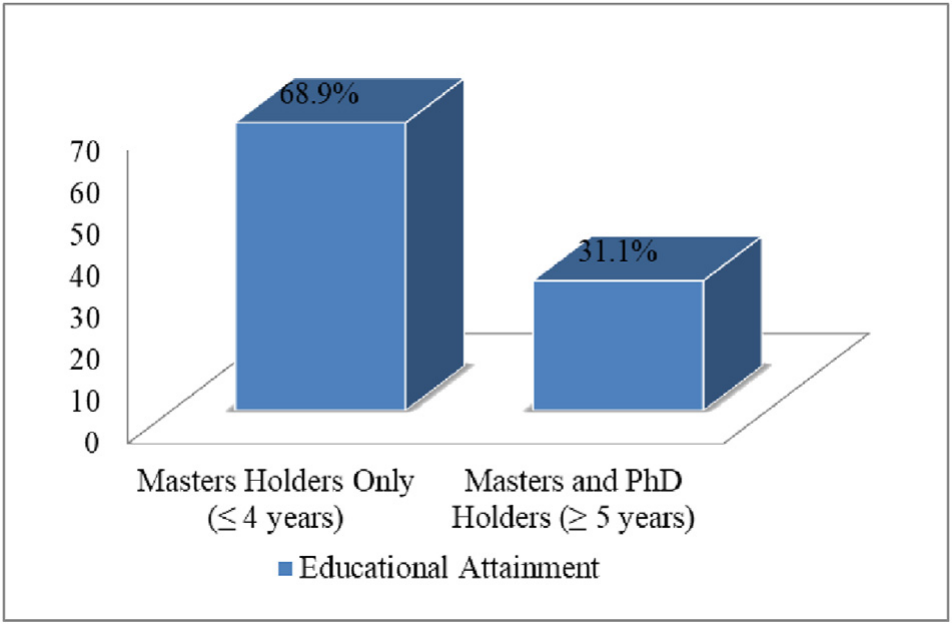
Bar Chart showing the educational attainment of the respondents. Source: Field Survey (2020).

Figure 2 displays respondents' educational attainment, 68.9% have spent less than 4 years to obtain a masters’ degree while the remaining spent more than 5 years to get masters and Doctoral degrees.

The result of the regression in Table 3 indicates a strong correlation between the educational qualification of the respondents and compliance with e-learning (R = 0.853^a^ > 0.51 for 180 degrees of freedom). The F-statistics (F = 475.356, p = 0.000^c^) is high and significant which indicates a strong influence of education on compliance with e-learning. The coefficient of R^2^ (0.728^b^) shows that 72.8% variation in e-learning compliance is caused by the educational attainment while the remaining 27.2% is attributed to the residual factors excluded from the regression model. Educational attainment (β = 0.843^d^) is positively significant at p < 0.05, that is, it has 84.3% influence on e-learning compliance. Hence, the educational attainment of the respondents is a strong predictor of e-learning compliance in Nigerian Private Tertiary Institutions. This implies that the educational attainment of the respondents could be harnessed and properly channeled towards e-learning compliance and sustainability.

**Table 3:** Correlation between respondents’ educational attainment and e-learning compliance.

Model	R	R^2^	Adjusted R^2^	Std. Error of the Estimate	Durbin-Watson
1	0.853^a^	0.728	0.726	2844.871	1.47
**ANOVA**^b^					
	**Sum of squares**	**df**	**Mean Square**	**F-Statistics**	**Sig.**
Regression	3.847E9	1	3.847E9	475.356	0.000^c^
Residual	1.441E9	178	8093293.014		
Total	5.288E9	179			
**Coefficients**					
**Model**	**Unstandardized Coefficients**		**Standardized Coefficients**		**Sig.**
	**β**	**Std. Error**	**Beta**	**T**	
(constant)	3134.401	611.235		5.128	0.000
Education Attainment	2252.060	103.293	0.843	21.803	0.000^d^

Source: Field Survey (2020).

a Predictor: (Constant), Education Attainment.

b Dependent variable: e-learning compliance.

c Predictor: (Constant), Education Attainment.

d Predictor: (Constant), Education Attainment.

**Figure 3:**
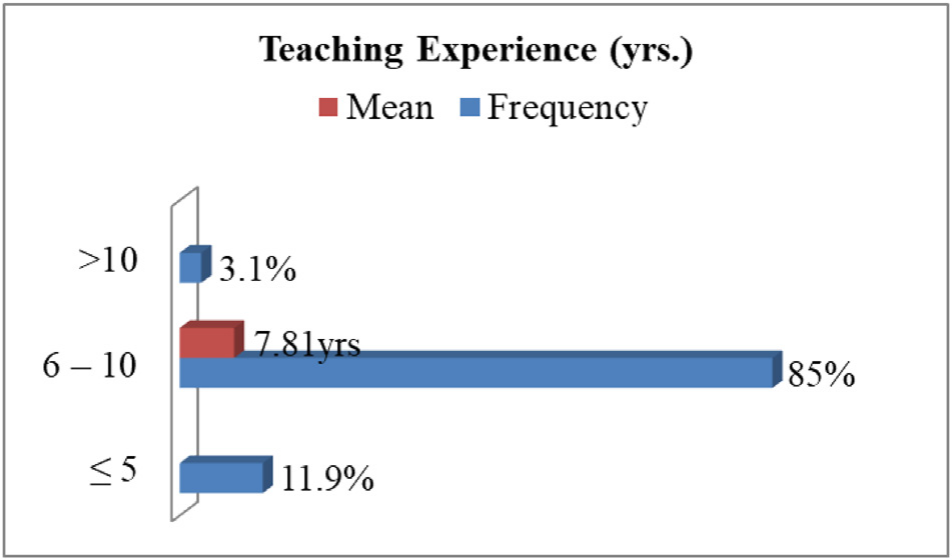
Bar Chart showing the distribution of respondents' teaching experience. Source: Field Survey (2020).

From the chart in Figure 3, the average teaching experience of the instructors was 7.8 years; 85% have joined the institutions since 6–10 years ago while only a few (3.1%) more than 10 years of experience in teaching.

A positive and weak correlation was revealed for years of experience in teaching and compliance with e-learning as shown in Table 4 (R = 0.169^a^ < 0.51 for 180 degrees of freedom). The F-statistics (F = 5.211, p = 0.024^c^) is significant but very low which further affirms that the relationship between years of experience in teaching and compliance to e-learning is weak. The coefficient of R^2^ (0.028^b^) shows that teaching experience is responsible for a 2.8% variation in e-learning compliance while the remaining 97.2% is attributed to the residual factors excluded from the regression model. There is a significant and positive relationship between teaching experience (β = 0.169^d^) and e-learning compliance at p < 0.05, this means that a 1% increase in the teaching experience would result in 16.9% compliance with e-learning. Hence, the teaching experience of the respondents influences e-learning compliance in Nigerian Private Tertiary Institutions. The implication for this study is that the instructors’ experience would be advantageous for capacity building and training on e-learning as little effort and lesser cost would be required to transmit the pedagogy and contents of e-learning to the instructors.

**Table 4:** Correlation between teaching experience of the respondents and e-learning compliance.

Model	R	R^2^	Adjusted R^2^	Std. Error of the Estimate	Durbin-Watson
1	0.169^a^	0.028	0.023	5372.322	1.472
**ANOVA**^b^					
	**Sum of squares**	**df**	**Mean Square**	**F-Statistics**	**Sig.**
Regression	1.504E8	1	1.504E8	5.211	0.024^c^
Residual	5.137E9	178	2.886E7		
Total	5.288E9	179			
**Coefficients**					
**Model**	**Unstandardized Coefficients**		**Standardized Coefficients**		**Sig.**
	**β**	**Std. Error**	**Beta**	**T**	
(constant)	13685.305	942.663		14.518	0.000
Teaching Experience	107.494	47.091	0.169	2.283	0.024^d^

Source: Field Survey (2020)

a Predictor: (Constant), Teaching Experience.

b Dependent variable: e-learning compliance.

c Predictor: (Constant), Teaching Experience.

d Predictor: (Constant), Teaching Experience.

### 3.2 Variance in compliance with the use of e-learning during COVID-19 lockdown in the Nigerian private tertiary institutions

**Figure 4:**
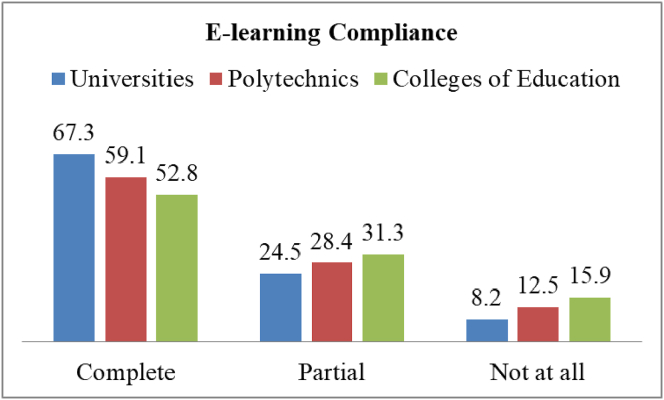
Bar Chart showing compliance to e-learning. Source: Field Survey (2020).

Figure 4 provides descriptive of e-learning compliance. The chart indicated that full compliance with e-learning was highest among the instructors in the private universities (67.3%), followed by the polytechnics (59.1%) and Colleges of Education (52.8%).

Results of ANOVA in Table 5 confirmed variation in the level of compliance with e-learning by the instructors in the private tertiary institutions (F = 15.36, p = 0.00). Results of Scheffe's post hoc test further showed that level of compliance with e-learning in the private Universities ($\bar{\text{X}}$ = 23.56), private Polytechnics ($\bar{\text{X}}$ = 22.05) and Colleges of Education ($\bar{\text{X}}$ = 21.97) were significantly different at p < 0.05. It reveals that Universities have the highest level of compliance, followed by Polytechnics and Colleges of Education. Universities have a comparative advantage in ICT gadgets than the Polytechnic and Colleges of Education in Nigeria. Nigerian private universities are at the forefront of e-learning implementation due to their innovative and flexible operations. Private universities have also embraced the e-learning platform to continue and sustain the academic calendar, and increase revenue generation to the school pulse. The implication is that Nigerian private universities would soon completely shift to digital education and able to offer courses beyond the shore of the country as it is being practiced in advanced countries. This will go a long way to improve the nation's education quality and standard, and ranking among the countries of the world.

**Table 5:** Test of difference in the level of e-learning compliance across the selected private tertiary institutions using ANOVA.

e-learning compliance	Sum of square	df	Mean square	F	Sig.	Scheffe's Post Hoc Tests
Between groups	182.51	2	91.25	15.36	0.00	Universities 23.5592^a^
Within groups	2881.16	177	5.94			Polytechnics 22.0469^b^
Total	3063.66	179				Colleges of Education 21.9740^c^

df – degree of freedom. a, b, c denote Scheffe’ contrast.

### 3.3 Relationship between the limitations and e-learning compliance

The linear regression in Table 6 has a coefficient of R^2^ of 0.73 indicating a 73% variation in the dependent is as a result of explanatory variables. Results in Table 6 indicated that challenges are strong determinants of compliance with e-learning. Significant relationships are found for poor power supply (β = -0.65), high cost and poor quality of e-learning facilities (β = -0.43), and poor technical know-how of e-learning (β = -0.62) at p < 0.05 level of significance. This is an indication that the power supply, e-learning facilities, and technical know-how of the instructors affected compliance by 65%, 43%, and 62% respectively. Also, there are significant but inverse relationships for poor internet connectivity (β = -0.78), lack of telecommunication infrastructure (β = -0.74), and lack of training support by the government (β = -0.83). It can be inferred that the limitations caused 71–83% non-compliance to the e-learning in the selected private tertiary institutions. So also, the significance of the F-value (F = 8.92) is a pointer to the fact that the relationship existed between the constraints and compliance to e-learning. It could be inferred that constraints retard e-learning development in the country and no tangible progress could be achieved in the education sector until these problems are addressed.

Linear: ϒ = α + (-0.65)L_1_ + (-0.43)L_2_ + (-0.62)L_3_ + (-0.78)L_4_+ (-0.74)L_5_+ (-0.83)L_6_ + ei

**Table 6:** Limitations and e-learning compliance.

Limitations	Βeta	Standard Error	T	Significant
Constant	4.16	0.30	13.87	0.00
Poor power supply	**-**0.65	0.14	**-**4.64	0.01∗
High cost and poor quality of ICT facilities	**-**0.43	0.05	**-**8.60	0.00∗
The poor technical know-how of ICT	**-**0.62	0.17	**-**3.65	0.01∗
Poor internet connectivity	**-**0.78	0.22	**-**3.55	0.01∗
Lack of telecommunication infrastructure	**-**0.74	0.23	**-**3.22	0.02∗
Lack of training support by the government	**-**0.83	0.12	**-**6.92	0.00∗
F – statistics	8.92			
R^2^	73.41			
Adjusted R	70.95			
Durbin-Watson	26.01			
Prob (F-Statistics)	0.00			

∗ – significant at p ≤ 0.05.

## 4 Discussion

Socio-economic variables influence compliance with e-learning. The age of the respondents has a significant relationship with e-learning compliance. The instructors are below forty years (mean = age of 36.8 years), which indicates the respondents are within the working-age population according to Hannah and Max (2019). Nigeria currently has 53.57% of her population in this bracket (Plecher, 2020) and they can learn new technology very fast, and adjust to electronic teaching. At this tender age, people are innovative and have a keen interest to learn new skills compared to people at old age. According to the Teaching and Learning International Survey (TALIS), younger teachers use technology more frequently in the classroom (Schleicher and Reimers, 2020). Plecher (2020) reported that the bracket would have an important impact on Nigeria's Educational Development. Also, experience counts in adaptation to new techniques of teachings. The correlation of teaching experience with e-learning compliance was positive and significant at p < 0.05. From the three selected socio-economic variables, the test of significance revealed that the educational attainment of the respondents has the greatest influence, a strong correlation and significantly predicts compliance to e-learning. Advanced education and ICT skills are particularly important given the radical shift towards online teaching during the COVID-19 lockdown (Andreas, 2020).

Compliance with the e-learning is high in the Nigerian universities when it compares to the situation in Polytechnics and Colleges, that is, compliance in the universities is encouraging. For the last two decades, private universities have outnumbered the private polytechnics and Colleges of Education. Both individuals and religious organizations invested much in private universities particularly in the southwest, Nigeria. Though the school fees at these universities are exorbitant they have good facilities for e-learning and a stable academic calendar. The Universities take into cognizance the importance of e-learning so, they are more proactive than the Polytechnics and Colleges. Eze et al. (2018), Mahmood (2020) and Ali (2020) opined that new inventions and technology give better ways of communication and interactions and it has tremendously increased knowledge. However, there exist limitations in the e-learning in the selected tertiary institutions. The problems have resulted in partial compliance to e-learning in the Polytechnics and Colleges of Education; the structural buildings and facilities in the Colleges and Polytechnics are very scanty to that of Universities –Libraries, Laboratories are ICT centers are well equipped. The shortage of electricity supply is persistent in Nigerian tertiary institutions and it usually distorts researches and studies. In a report of Thisday (2016), investment in power supply does not commensurate with the megawatt generated for use and it cannot go round. Likewise, Oyediran and Dick (2018) explained that the power supply to the public is diminishing and getting worst. Instructors that are Computer incline are very limited in many of these schools. Eze et al. (2018) argued that a lack of experts in ICT affects its use in Nigeria. In this technology age, e-learning is an essential mechanism of transferring knowledge and to fast-track academics transformation from traditional teaching to modern teaching in the Nigerian educational system.

## 5 Conclusion

This study established that socio-economic variables are significantly correlated with e-learning compliance with educational attainment as a major determinant. It was also found that variation existed in e-learning compliance across the selected private tertiary institutions, a pointer to the fact that e-learning has not been effectively incorporated into tertiary education in Nigeria; the private universities have the highest level of compliance with e-learning during the COVID-19 pandemic. The limitations obstruct compliance to e-learning particularly in the private Polytechnics and Colleges of Education in the southwest, Nigeria and it would have multiply effect on the academic progress of the institutions and could further create a socio-economic skills gap for the nation. Regression analysis affirmed the significance and negative influence of constraints on the instructors' compliance to e-learning in the selected private tertiary institutions at p < 0.05. The implication for this study is that instructors' SEV and limitations could undermine e-learning compliance during and after the pandemic in Nigeria. Globally, e-learning has been identified as an indispensable intervention to cushion the impact of the COVID-19 pandemic and as well for rapid growth and development in the education sector of any nation. The advantages of e-learning include wide coverage, cost-effectiveness, uniformity, fast teaching and learning process, and rapid economic development through e-commerce. It is hereby recommended that compliance to e-learning in the tertiary institutions should go beyond the COVID-19 lockdown period while staff training and capacity building on e-learning should be put in place by the institutions’ authority. The government should address challenges limiting e-learning in the tertiary institutions through the provision of stable power supply, and local industries should be encouraged to manufacture some ICT accessories to lessen the cost of acquisition arising from a high tariff. These recommendations become very important going by the rapidly changing world of basic education through digitization.

## Declarations

### Author contribution statement

W. O. Oyediran: Conceived and designed the experiments; Analyzed and interpreted the data; Wrote the paper.

A. M. Omoare: Performed the experiments; Analyzed and interpreted the data; Wrote the paper.

M. A. Owoyemi, R. B. Fasasi: Performed the experiments; Contributed reagents, materials, analysis tools or data.

A. O. Adejobi: Contributed reagents, materials, analysis tools or data.

### Funding statement

This research did not receive any specific grant from funding agencies in the public, commercial, or not-for-profit sectors.

### Declaration of interests statement

The authors declare no conflict of interest.

### Additional information

No additional information is available for this paper.
